# Serum 25-hydroxyvitamin D might be negatively associated with hyperuricemia in U.S. adults: an analysis of the National Health and Nutrition Examination Survey 2007–2014

**DOI:** 10.1007/s40618-021-01637-x

**Published:** 2021-08-26

**Authors:** Y. Han, K. Han, Y. Zhang, X. Zeng

**Affiliations:** 1 Department of General Internal Medicine, Peking Union Medical College Hospital, Chinese Academy of Medical Sciences, No. 1 Shuaifuyuan, Dongcheng District, Beijing, 100730 China; 2 Department of Critical Care Medicine, The Affiliated Yantai Yuhuangding Hospital of Qingdao University, Yantai, 264000 Shandong China

**Keywords:** Uric acid, 25-hydroxyvitamin D, Vitamin D, Hyperuricemia, NHANES

## Abstract

**Purpose:**

The results of previous studies on the relationship between serum 25-hydroxyvitamin D [25(OH)D] and hyperuricemia are controversial. We hypothesized that serum 25(OH)D concentrations of U.S. adults would negatively correlate with the risk of hyperuricemia.

**Method:**

Data came from the National Health and Nutrition Examination Survey 2007–2014 were used, after excluding those who met at least one of the exclusion criteria, a total of 9096 male individuals and 9500 female individuals aged 18 years or older were included. Binary logistic regression analysis and restricted cubic spline with fully adjusted confounding factors were applied to evaluate the association between serum 25(OH)D and hyperuricemia. We further performed stratified analysis and sensitivity analysis to minimize the influence of gender, metabolic syndrome, obesity and renal dysfunction on the above association.

**Results:**

We found a negative correlation between serum 25(OH)D and hyperuricemia. In the binary logistic regression analysis, compared with the highest serum 25(OH)D quartile [Q4: 25(OH)D > 77.10 nmol/L] group, the odds ratios (95% confidence intervals) in the lowest quartile [Q1: 25(OH)D ≤ 43.20 nmol/L] was 1.46 (1.22–1.75) in the fully adjusted model. Restricted cubic spline analysis showed L-shaped and non-linear relationships between 25(OH)D and hyperuricemia. In sensitivity analysis, after restricting to participants without significant renal dysfunction and obesity, the above association remained significant. After restricting to participants who were diagnosed as metabolic syndrome, above association remained significant in the fully adjusted model. In stratified analysis by gender, the association remained significant among males and females.

**Conclusions:**

Serum 25(OH)D might be inversely associated with hyperuricemia in general U.S. adults. From our study, for people with unexplained hyperuricemia, screening for serum Vitamin D concentration might be necessary.

## Introduction

Vitamin D from both endogenous synthesis by skin and exogenous intake from diet are metabolized in the liver to 25-hydroxyvitamin D [25(OH)D], which is the most widely used marker and estimator of Vitamin D status. An epidemiological investigation in National Health and Nutrition Examination Survey (NHANES) has shown the overall shift of the population toward lower serum 25(OH)D concentrations and higher prevalence of hypovitaminosis D [1]. Previous study has found that 18.3% of US noninstitutionalized population were at a risk of Vitamin D inadequacy and 5% were at a risk of deficiency [2]. Vitamin D plays an important role in regulating the normal homeostasis of calcium and phosphorus. Adequate concentrations of Vitamin D are necessary for bone health, while low concentrations of Vitamin D are associated with rickets and osteomalacia [2]. In addition, Vitamin D has many non-calcemic biological functions. Experimental studies indicate that Vitamin D, as a negative regulator of renin–angiotensin system, can suppress renin expression [3]. Furthermore, Vitamin D maintains pancreatic β cells’ function through binding of vitamin D with vitamin D receptor, transduction of insulin signaling and engaging in regulation of Ca^2+^ flux in the pancreatic β cells [4]. Vitamin D is also associated with cell proliferation and differentiation and can inhibit the growth of cancers in experimental animals [5]. Observational studies have revealed the association between Vitamin D deficiency and a variety of clinical conditions, such as poor blood pressure control, risk of hypertension [6, 7], cardiovascular morbidity and mortality [8], higher fasting and postprandial blood glucose in type 2 diabetes mellitus patients [9], dyslipidemia [10] and autoimmune disease [11]. Even if the evaluation of the circulating levels of 25(OH)D in these categories of subjects per se does not justify the supplementation of vitamin D for the therapeutic purpose at the moment, controversial results in this regard have been published and there is no general consensus on these topics [12–15].

Uric acid is the end product of purine nucleotide metabolism and hyperuricemia can occur as a result of overproduction or underexcretion of serum uric acid (SUA), but the pathophysiological mechanism of hyperuricemia has not yet been fully elucidated. SUA has a protective effect in neurodegenerative disease, such as dementia, Parkinson's disease [16]; however, hyperuricemia is a common health problem which affects nearly 21% of US adults [17]. In addition to joint involvement, hyperuricemia has been linked with different degrees of some metabolic disorders [18–21], including hypertension, obesity, type 2 diabetes and metabolic syndrome. Numerous epidemiological studies have shown that increased SUA is an independent risk factor for cardiovascular and all-cause mortality [22, 23].

Previous studies have demonstrated the association between SUA and Vitamin D, but the results are inconsistent and controversial. Some studies have pointed out that 25(OH)D insufficiency is significantly associated with increased odds ratios (ORs) of elevated SUA [24, 25], which is consistent with our results. However, other studies have come to different conclusions [26, 27]. We hypothesized that serum 25(OH)D concentrations of U.S. adults would negatively correlate with the risk of hyperuricemia. Binary logistic regression analysis and restricted cubic spline with fully adjusted confounding factors were applied to evaluate the association between serum 25(OH)D concentrations and hyperuricemia. Therefore, we performed this study in a large, nationally representative cohort of U.S. adults using data from the NHANES 2007–2014 to explore whether there was a negative correlation between vitamin D and hyperuricemia and hoped it can provide some information for the treatment of hyperuricemia.

## Materials and methods

### Data source and study population

The NHANES, which was conducted by the Centers for Disease Control and Prevention of America, collected information regarding the health and nutritional status of the U.S. population every 2 years. NHANES used a complex, stratified sampling design, which can select representative samples of non-institutionalized civilians. Participants underwent a detailed in-home interview and a physical examination and blood specimen collection at specially equipped mobile examination centers [28]. The study was conducted in accordance with the Declaration of Helsinki [29]. Written informed consent was obtained from all participants prior to completing the NHANES, and all data were de-identified by the National Center for Health Statistics before being made publicly available.

**Fig. 1:**
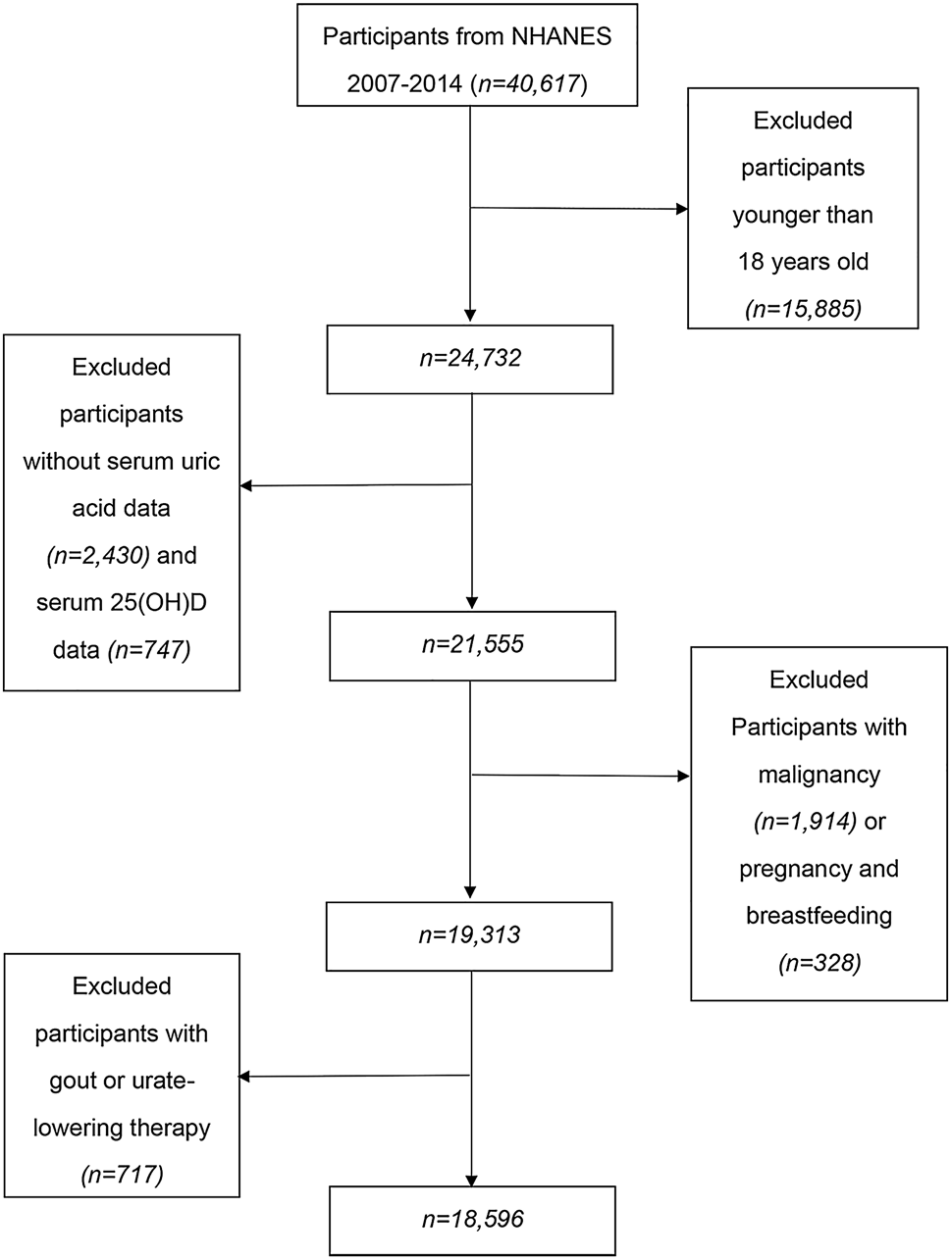
Flow chart of the screening process for the selection of eligible participants

Participants for the present analysis consisted of individuals enrolled in four 2-year cycle surveys conducted in 2007–2008, 2009–2010, 2011–2012 and 2013–2014. A total of 40,617 adult participants enrolled in the NHANES from 2007 to 2014, and the exclusion criteria were to meet one of the following: (1) age < 18 years (*n* = 15,885); (2) participants whose serum uric acid data (*n* = 2,430) and serum 25(OH)D data (*n* = 747) were missing; (3) self-reported cancer or malignancy (*n* = 1914); (4) being pregnant or breastfeeding (*n* = 328); (5) self-reported gout or received urate-lowering therapy (*n* = 717). Eventually, a large national representative sample (9096 males, 9500 females) among the general adult US population was included in this study. The flow chart of the screening process is shown in Fig. 1.

### Study variables

Serum 25(OH)D was used as a direct measure of Vitamin D status, which reflected cumulative endogenous synthesis and exogenous supplement. Serum 25-hydroxyvitamin D3 and 25-hydroxyvitamin D2 are measured by an ultra-high performance liquid chromatography-tandem mass spectrometric method [30]. The ultra-high performance liquid chromatography-tandem mass spectrometric (LC–MS) method has improved sensitivity and specificity for serum 25(OH)D metabolites, and the standardization of serum 25(OH)D data allows for comparison across different survey cycles of the NHANES, providing sufficient power to study risk associated with varying concentrations of serum 25(OH)D [31]. In addition, the preparation of reagents and quality control materials for the LC–MS method are standardized; previous study found no significant differences between the 2-year survey cycles [32]. Total 25(OH)D is the sum of 25-hydroxyvitamin D2 and 25-hydroxyvitamin D3. Serum uric acid is measured by DxC800 synchron using a timed endpoint method [33]. Hyperuricemia is defined as serum uric acid level ≥ 420 μmol/L (7 mg/dL) and ≥ 360 μmol/L (6 mg/dL) in males and females, respectively [34].

Potential confounding factors in this study include age, gender, race (Mexican American, Other Hispanic, Non-Hispanic White, Non-Hispanic Black and Other race), body mass index (BMI) (Normal: between 18.5 and 25 kg/m^2^; overweight: between 25 and 30 kg/m^2^; obesity: ≥ 30 kg/m^2^) [35], waist circumference, estimation of the glomerular filtration rate [eGFR is calculated according to the Modification of Diet in Renal Disease equation, eGFR = 175 × standardized Scr^−1.154^ × age^−0.203^ × 1.212 (if black) × 0.742 (if female)] [36], and significant chronic kidney dysfunction is defined as decreased eGFR less than 60 mL/min/1.73 m^2^ [37]. Poverty income ratio (PIR) was used to define income, which was categorized as less than 0.99 and 1 or more. A PIR lower than 1.0 represents a person is living under poverty line. Serum calcium, phosphorus, total cholesterol, triglyceride, smoking status (smoked at least 100 cigarettes in life or not) and drinking status (had at least 12 alcohol drinks/1 year) were also involved in this study. The history of hypertension, diabetes was defined as participants self-reported diagnosis of hypertension or diabetes by physicians. Degree of physical activity (Physically active group, insufficiently active group and inactive group) was also involved in this study.

### Statistical analysis

All statistical analyses were conducted with R 4.0.2. To account for the complex sampling design and ensure nationally representative estimates, all analyses were adjusted for survey design and weighting variables. New sample weight (the original 2-year sample weight divided by 4) was constructed according to the analytical guidelines of the NHANES [38]. The normality of continuous variables was tested with Kolmogorov–Smirnov normality test. Normally distributed variables were described with mean ± standard deviation, and non-normally distributed continuous variables were described with median (interquartile range). The median values among different serum 25(OH)D and SUA groups were compared with the Mann–Whitney *U* test and Kruskal–Wallis test. The χ^2^ test was adopted to compare the percentages of categorical variables among different serum 25(OH)D and SUA groups. The Bonferroni test was used for the intergroup comparison. Serum 25(OH)D level in binary logistic regression analyses were modeled in quartiles: Q1 (25(OH)D ≤ 43.20 nmol/L), Q2 (43.20 < 25(OH)D ≤ 59.70 nmol/L), Q3 (59.70 < 25(OH)D ≤ 77.10 nmol/L) and Q4 (25(OH)D > 77.10 nmol/L), with the fourth quartile (Q4) as the reference group. Binary logistic regression analysis was conducted to examine the association between 25(OH)D and SUA. Age and gender were adjusted in model 1, and model 2 was additionally adjusted for race, BMI, waist circumference, PIR, drinking status, smoking status, physical activity, hypertension, diabetes, total cholesterol, triglyceride, eGFR, serum calcium and phosphorus. Restricted cubic spline analysis with 3 knots of the vitamin D concentration was used to characterize the dose–response relationship in the logistic regression Model 2. To exclude the influence of renal dysfunction and obesity, we then performed a sensitivity analysis by restricting to participants without chronic kidney disease and obesity participants. In addition, gender stratified analysis was performed to examine above association. We also performed above analysis in participants who were diagnosed as metabolic syndrome. Participants who with at least three of the five components (hypertension, diabetes, abdominal obesity, hypertriglyceridemia, or low levels of HDL) were diagnosed as metabolic syndrome [39]. In our study, quartiles of serum vitamin D were used to explore the relationship between vitamin D and SUA, and we used restricted cubic splines to characterize the dose–response relationship between vitamin D and hyperuricemia for the first time. A two-sided *P* < 0.05 was considered statistically significant.

## Results

A total of 18,596 individuals (9096 men, 9500 women) were involved in our study. All participants were categorized into five racial groups: Mexican American (*n* = 3067), Other Hispanic (*n* = 2026), Non-Hispanic White (*n* = 7642), Non-Hispanic Black (*n* = 3850), Other race (*n* = 2011). The mean age was 46.02 ± 17.95 years, the mean serum 25(OH)D level was 62.17 ± 26.50 nmol/L and mean SUA level was 321.75 ± 83.19 μmol/L. 17.10% of the participants met the diagnostic criteria of hyperuricemia and 12.21% of the participants had a SUA concentration ≥ 420 μmol/L. The clinical characteristics of participants with different serum Vitamin D levels are shown in Table 1. We found a greater proportion of hyperuricemia participants that belonged to the lowest quartile of Vitamin D level. The two lower 25(OH)D quartiles showed larger waist circumference than the two higher quartiles. With the increase in 25(OH)D quartiles, the proportion of physically active participants increased gradually, while the median of BMI, eGFR and the proportion of people living under poverty line declined gradually. The trend of eGFR in our research was consistent with the study conducted by Seong-Woo Choi, and the reduced 25(OH)D clearance in participants with lower eGFR might be the possible mechanism [40, 41].

**Table 1:** Clinical characteristics of the study population in disaggregated by quartiles of serum 25(OH)D level. NHANES 2007–2014 (*N* = 18,596)

Serum 25(OH)D quartile	Q1	Q2	Q3	Q4	*P* value
Number of subjects	4653	4651	4644	4648	
Age (year)^b^	41 (27)	43 (28)	45 (28)	52 (30)	< 0.01
Gender (%)^a^					< 0.01
Male	2145 (46.1)	2489 (53.5)	2493 (53.7)	1969 (42.4)	
Female	2508 (53.9)	2162 (46.5)	2151 (46.3)	2679 (57.6)	
Race (%)^a^					< 0.01
Mexican American	898 (19.3)	1047 (22.5)	762 (16.4)	360 (7.7)	
Other Hispanic	437 (9.4)	664 (14.3)	558 (12.0)	367 (7.9)	
Non-Hispanic White	786 (16.9)	1504 (32.3)	2274 (49.0)	3078 (66.2)	
Non-Hispanic Black	1960 (42.1)	888 (19.1)	571 (12.3)	431 (9.3)	
Other race	572 (12.3)	548 (11.8)	479 (10.3)	412 (8.9)	
Physical activity (%)^a^					< 0.01
Inactive	2734 (58.8)	2422(52.1)	2317 (49.9)	2066 (44.4)	
Insufficiently active	638(13.7)	669 (14.4)	705 (15.2)	745 (16.0)	
Physically active	1281 (27.5)	1560 (33.5)	1622 (34.9)	1837 (39.5)	
Waist circumstance (cm)^b^	98.1 (23.9)	97.6 (20.8)	96.2 (20.1)	93.4 (21.7)	< 0.01
Body mass index (kg/m^2^)^b^	29.0 (9.9)	28.3 (8.0)	27.4 (7.3)	26.3 (7.1)	< 0.01
Cholesterol (mmol/L)^b^	4.81 (1.45)	4.86 (1.42)	4.91 (1.40)	4.94 (1.45)	< 0.01
Triglyceride (mmol/L)^b^	1.22(1.13)	1.36 (1.26)	1.37 (1.23)	1.33 (1.14)	< 0.01
eGFR (mL/min/1·73 m^2^)^b^	99.5(32.8)	95.4 (30.5)	89.7(28.0)	81.6 (26.1)	< 0.01
Poverty income ratio < 1 (%)^a^	1247 (29.4)	1116 (26.4)	958 (22.4)	786 (18.4)	< 0.01
Hyperuricemia (%)^a^	897 (19.3)	798 (17.2)	690 (14.9)	794 (17.1)	< 0.01
Serum 25(OH)D (nmol/L)^b^	33.0 (11.8)	51.9 (8.3)	67.5 (8.5)	90.8 (20.8)	< 0.01
Total calcium(mmol/L)^b^	2.35 (0.125)	2.35 (0.100)	2.35 (0.100)	2.38 (0.100)	< 0.01
Phosphorus(mmol/L)^b^	1.20 (0.226)	1.23 (0.226)	1.23 (0.226)	1.23 (0.258)	< 0.01
Hypertension (%)^a^	1373 (29.5)	1330 (28.6)	1377 (29.7)	1690 (35.4)	< 0.01
Diabetes (%)^a^	507 (10.9)	509 (11.0)	456 (9.8)	489 (10.5)	0.26
Had at least 12 alcohol drinks/year (%)^a^	2740 (68.2)	2876 (70.2)	3103 (74.9)	3119 (74.5)	< 0.01
Smoked at least 100 cigarettes in life (%)^a^	1868 (42.5)	1816 (41.0)	1999 (44.5)	2035 (45.2)	< 0.01

Data are number of subjects (percentage) or medians (interquartile ranges)

*Serum 25(OH)D quartiles* Q1 (25(OH)D ≤ 43.20 nmol/L), Q2 (43.20 < 25(OH)D ≤ 59.70 nmol/L), Q3 (59.70 < 25(OH)D ≤ 77.10 nmol/L), Q4 (25(OH)D > 77.10 nmol/L).

^a^χ^2^ test was used to compare the percentage among participants in different groups

^b^Kruskal–Wallis test was used to compare the median values among participants in different groups

The clinical characteristics of participants with different SUA levels are shown in Table 2. People with hyperuricemia were older (52 vs. 44 years old) and have higher serum calcium (2.38 vs. 2.35 mmol/L), triglyceride (1.67 vs. 1.25 mmol/L), higher BMI (30.94 vs. 27.02 kg/m^2^) and waist circumference (105.6 vs. 94.4 cm), and higher prevalence of hypertension (50.3 vs. 27.1%) and diabetes (16.0 vs. 9.4%). Participants in normal SUA level group are younger and physically active, more likely to be females and have higher eGFR (93.42 vs. 79.21 mL/min/1.73 m^2^).

**Table 2:** Clinical characteristics of the study population in hyperuricemia group and normal serum uric acid group. NHANES 2007–2014 (*N* = 18,596)

	Normal serum uric acid	Hyperuricemia	*P* value
Number of subjects	15,417	3179	
Age (year)^b^	44 (29)	52 (31)	< 0.01
Gender^a^			< 0.01
Male (%)	7333 (47.6)	1763 (55.5)	
Female (%)	8084 (52.4)	1416 (44.5)	
Race (%)^a^			< 0.01
Mexican American	2682 17.4)	385 (12.1)	
Other Hispanic	1772 11.5)	254 (8.0)	
Non-Hispanic White	6251 (40.5)	1391 (43.8)	
Non-Hispanic Black	3039 19.7)	811 (25.5)	
Other race	1673 (10.9)	338 (10.6)	
Physical activity (%)^a^			< 0.01
Inactive	7740 (50.2)	1799 (56.6)	
Insufficiently active	2302 (14.9)	455 (14.3)	
Physically active	5375 (34.9)	925 (29.1)	
Waist circumstance (cm)^b^	94.4 (20.9)	105.6 (20.4)	< 0.01
Body mass index (kg/m^2^)^b^	27.02 (7.77)	30.94 (9.1)	< 0.01
Cholesterol (mmol/L)^b^	4.86 (1.40)	5.02 (1.47)	0.811
Triglyceride (mmol/L)^b^	1.25 (1.12)	1.67 (1.40)	< 0.01
eGFR (mL/min/1·73 m^2^)^b^	93.42 (29.71)	79.21 (32.40)	< 0.01
Poverty income ratio < 1 (%)^a^	3445 (24.4)	662 (22.8)	0.064
Serum uric acid (μmol/L)^b^	297.4 (95.2)	440.2 (65.4)	< 0.01
Serum 25(OH)D (nmol/L)^b^	60.1 (33.45)	58.0 (36.3)	< 0.01
Total calcium(mmol/L)^b^	2.35 (0.100)	2.38 (0.125)	< 0.01
Phosphorus(mmol/L)^b^	1.23 (0.226)	1.23 (0.226)	0.892
Hypertension (%)^a^	4172 (27.1)	1598 (50.3)	< 0.01
Diabetes (%)^a^	1452 (9.4)	509 (16.0)	< 0.01
Had at least 12 alcohol drinks/year (%)^a^	9780 (72.1)	2058 (71.5)	0.53
Smoked at least 100 cigarettes in life (%)^a^	6267 (42.6)	1451 (46.9)	< 0.01

Data are number of subjects (percentage) or medians (interquartile ranges)

^a^χ^2^ test was used to compare the percentage between participants with and without hyperuricemia

^b^Mann–Whitney *U* test was used to compare the median values between participants with and without hyperuricemia

**Fig. 2:**
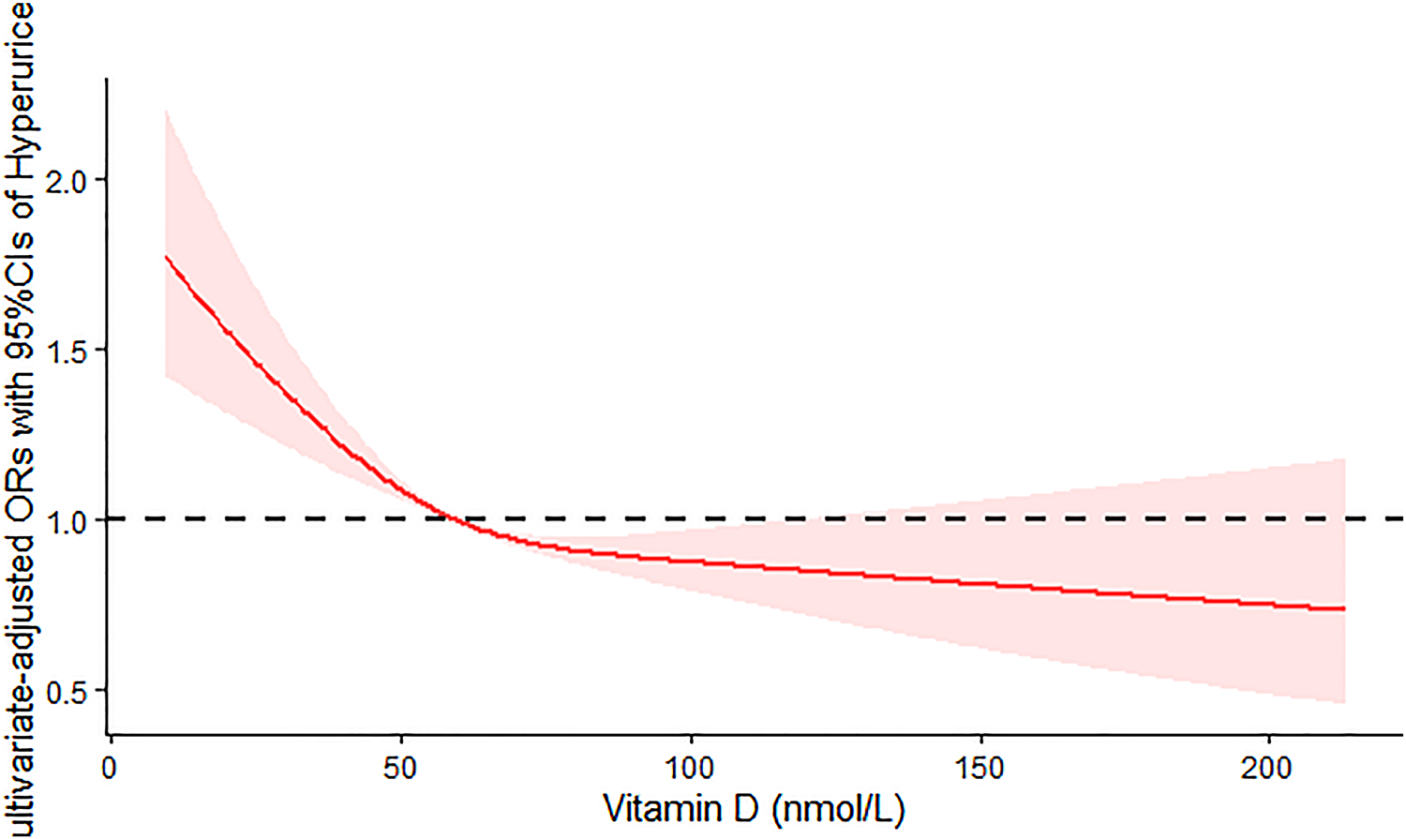
Examination of the dose–response relationship between serum 25(OH)D (nmol/L) and the risk of hyperuricemia by restricted cubic splines model. The restricted cubic splines model adjusted for age, gender, race, BMI: waist circumference, PIR: physical activity, drinking status, smoking status, hypertension, diabetes, total cholesterol, triglyceride, eGFR: serum calcium and phosphorus

The results of binary logistic regression analysis between serum 25(OH)D levels and hyperuricemia are presented in Table 3. In Table 3, the crude ORs with 95% confidence intervals (CIs) of hyperuricemia are 0.95 (0.82–1.09), 1.16 (0.98–1.37) and 1.33 (1.14–1.55) in Q3, Q2 and Q1 versus Q4 of 25(OH)D, respectively. In Model 1, after adjustment for age and gender, the adjusted ORs with 95% CI are 0.97 (0.84–1.11), 1.23 (1.04–1.46) and 1.49 (1.28–1.73) in Q3, Q2 and Q1 versus Q4 of 25(OH)D, respectively. In Model 2, the multivariate-adjusted ORs with 95% CIs of hyperuricemia are 0.96 (0.81–1.14), 1.14 (0.94–1.38) and 1.46 (1.22–1.75) in Q3, Q2 and Q1 versus Q4 of serum 25(OH)D, respectively. The results of the restricted cubic spline dose–response relationship analysis between serum 25(OH)D and hyperuricemia were presented in Fig. 2. We found a suggestion of L-shaped associations of serum 25(OH)D concentration and hyperuricemia. The prevalence of hyperuricemia decreased with increasing serum 25(OH)D concentration and showed a nonlinear dose–response relationship (*P* non-linearity = 0.0031).

**Table 3:** Weighted odds ratios (95% confidence intervals) for hyperuricemia of participants across quartiles of serum 25(OH)D, NHANES 2007–2014(*N* = 18,596)

Serum 25(OH)D quartiles	Case/Participants	Crude^a^	Model 1^a^	Model 2^a^
Q4	4648/18596	1.00 (Ref.)	1.00 (Ref.)	1.00 (Ref.)
Q3	4644/18596	0.95 (0.82–1.09)	0.97 (0.84–1.11)	0.96 (0.81–1.14)
Q2	4651/18596	1.16 (0.98–1.37)	1.23 (1.04–1.46)*	1.14 (0.94–1.38)
Q1	4653/18596	1.33 (1.14–1.55)**	1.49 (1.28–1.73)**	1.46 (1.22–1.75)**

*Serum 25(OH)D quartiles* Q1 (25(OH)D ≤ 43.20 nmol/L), Q2 (43.20 < 25(OH)D ≤ 59.70 nmol/L), Q3 (59.70 < 25(OH)D ≤ 77.10 nmol/L), Q4 (25(OH)D > 77.10 nmol/L), *Model 1* adjusted for age and sex, *Model 2* adjusted for age, sex and race, *BMI* waist circumstance, poverty income ratio, physical activity, drinking status, smoking, hypertension, diabetes, total cholesterol, triglyceride, *eGFR* calcium, phosphorus

^a^Calculated using binary logistic regression

*P < 0.05, **P < 0.01

Additionally, we further performed sensitivity analysis by excluding participants with significant renal dysfunction (eGFR lower than 60 mL/min/1.73 m^2^) and obesity participants (BMI ≥ 30 kg/m^2^); 11,006 participants were involved in the sensitivity analysis, and after fully adjusting the same confounding factors, the associations between serum 25(OH)D and hyperuricemia are still significant (Table 4).

**Table 4:** Weighted odds ratios (95% confidence intervals) for hyperuricemia of participants without chronic kidney disease and obesity across quartiles of serum 25(OH)D level, NHANES 2007–2014 (*N* = 11,006)

Serum 25(OH)D quartiles	Case/Participants	Crude^a^	Model 1^a^	Model 2^a^
Q4	2750/11006	1.00 (Ref.)	1.00 (Ref.)	1.00 (Ref.)
Q3	2750/11006	1.06 (0.87–1.30)	0.96 (0.79–1.18)	1.00 (0.77–1.30)
Q2	2746/11006	1.14 (0.90–1.45)	1.05 (0.82–1.34)	1.08 (0.78–1.48)
Q1	2760/11006	1.27 (1.01–1.60)*	1.23 (0.97–1.56)	1.43 (1.06–1.93)*

*Serum 25(OH)D quartiles* Q1 (25(OH)D ≤ 45.50 nmol/L), Q2 (45.50 < 25(OH)D ≤ 61.80 nmol/L), Q3 (61.80 < 25(OH)D ≤ 78.90 nmol/L), Q4 (25(OH)D > 78.90 nmol/L), *Model 1* adjusted for age and sex, *Model 2* adjusted for age, sex and race, *BMI* waist circumstance, poverty income ratio, physical activity, drinking status, smoking, hypertension, diabetes, total cholesterol, triglyceride, *eGFR* serum calcium and phosphorus

^a^Calculated using binary logistic regression

*P < 0.05, **P < 0.01

In stratified analyses by gender, the results are shown in Table 5. Serum 25(OH)D levels were negatively associated with the risk of hyperuricemia in both men group and women group, and the adjusted ORs with 95%CIs were 1.30 (1.03–1.65) and 1.55 (1.13–2.12) in model 2, respectively.

**Table 5:** The weighted odds ratios (95% confidence intervals) of hyperuricemia across quartiles of serum 25(OH)D level, stratified analysis by gender, NHANES 2007–2014 (*N* = 18,596)

25(OH)D quartiles	Men			Women		
	Crude^a^	Model 1^a^	Model 2^a^	Crude^a^	Model 1^a^	Model 2^a^
Q4	1.00 (Ref.)	1.00 (Ref.)	1.00 (Ref.)	1.00 (Ref.)	1.00 (Ref.)	1.00 (Ref.)
Q3	0.91 (0.76–1.10)	0.91 (0.76–1.10)	0.92 (0.72–1.16)	0.89 (0.71–1.11)	1.09 (0.88–1.34)	1.07 (0.82–1.39)
Q2	1.15 (0.93–1.41)	1.15 (0.93–1.42)	1.07 (0.82–1.41)	1.03 (0.83–1.29)	1.37 (1.09–1.72) **	1.23 (0.94–1.62)
Q1	1.28 (1.04–1.58) *	1.28 (1.04–1.58) *	1.30 (1.03–1.65) *	1.34 (1.06–1.70) *	1.79 (1.43–2.25) **	1.55 (1.13–2.12) **

*Serum 25(OH)D quartiles* Q1 (25(OH)D ≤ 43.20 nmol/L), Q2 (43.20 < 25(OH)D ≤ 59.70 nmol/L), Q3 (59.70 < 25(OH)D ≤ 77.10 nmol/L), Q4 (25(OH)D > 77.10 nmol/L), *Model 1* adjusted for age, *Model 2* adjusted for age, race, *BMI* waist circumstance, poverty income ratio, physical activity, drinking status, smoking, hypertension, diabetes, total cholesterol, triglyceride, *eGFR* serum calcium and phosphorus

^a^Calculated using binary logistic regression

*P < 0.05, **P < 0.01

Finally, we performed logistic regression analysis in participants who were diagnosed with metabolic syndrome and the results are presented in Table 6. Serum 25(OH)D levels are negatively associated with the risk of hyperuricemia in model 2; the adjusted ORs with 95% CIs was 1.49 (1.16–1.91) in Q1 versus Q4 of serum 25(OH)D.

**Table 6:** Weighted odds ratios (95% confidence intervals) for hyperuricemia of participants who were diagnosed with metabolic syndrome, NHANES 2007–2014 (*N* = 6022)

Serum 25(OH)D quartiles	Case/Participants	Crude^a^	Model 1^a^	Model 2^a^
Q4	1499/6022	1.00 (Ref.)	1.00 (Ref.)	1.00 (Ref.)
Q3	1501/6022	0.84 (0.69–1.02)	0.86 (0.71–1.04)	1.06 (0.86–1.31)
Q2	1516/6022	0.95 (0.78–1.16)	0.99 (0.82–1.21)	1.16 (0.94–1.44)
Q1	1506/6022	1.07 (0.86–1.32)	1.12 (0.91–1.39)	1.49 (1.16–1.91) **

*Serum 25(OH)D quartiles* Q1 (25(OH)D ≤ 43.20 nmol/L), Q2 (43.20 < 25(OH)D ≤ 59.70 nmol/L), Q3 (59.70 < 25(OH)D ≤ 77.10 nmol/L), Q4 (25(OH)D > 77.10 nmol/L), *Model 1* adjusted for age and sex, *Model 2* adjusted for age, sex and race, BMI, waist circumstance, poverty income ratio, physical activity, drinking status, smoking, hypertension, diabetes, total cholesterol, triglyceride, *eGFR* serum calcium and phosphorus

^a^Calculated using binary logistic regression

*P < 0.05, **P < 0.01

## Discussion

In this study, we combined data from the NHANES 2007–2014 to investigate the association; a total of 9096 men and 9500 women aged 18 years or older were included. In binary logistic regression analysis, we found that serum 25(OH)D might be negatively associated with the risk of hyperuricemia. The association between serum 25(OH)D and hyperuricemia remained significant in both sensitivity analyses and stratified analyses. Restricted cubic spline found a non-linear relationship and L-shaped dose–response relationship between serum 25(OH)D and hyperuricemia.

The mechanisms of the relationship between Vitamin D and hyperuricemia remain unclear; several possible explanations have been proposed. Primarily, Vitamin D deficiency can cause secondary hyperparathyroidism, which leads to an increase of the serum parathyroid hormone (PTH) concentration [42]. Except for Vitamin D deficiency, daily calcium intake also affects the level of PTH; reduced intake of calcium is associated with high levels of serum PTH while excessive intake of calcium lowers PTH [43, 44]. Previous studies have shown a positive correlation between PTH and SUA and have suggested that PTH can affect the secretion and transport of uric acid [37, 45, 46], but the detailed mechanism is not clearly explained yet. Additionally, both laboratory studies and observational studies have revealed the association between hypovitaminosis D and insulin resistance [4, 47, 48] and vitamin D supplementation has a beneficial effect on insulin sensitivity [4, 49], while insulin resistance is inversely correlated to the renal clearance of SUA and can lead to hyperuricemia [18]. Insulin resistance is a major underlying mechanism for the metabolic syndrome, which is often complicated with hyperuricemia. Third, hyperuricemia and obesity are significantly correlated, while low serum Vitamin D is common in obese people due to volumetric dilution into greater volume of serum, fat [50] and less sunlight exposure [51]. Obesity-related adipose tissue inflammation and resulting dysfunction might be the central mechanism for the development of insulin resistance and metabolic diseases, both of which are associated with hypovitaminosis D and hyperuricemia [52, 53]. In addition, dysfunctional adipose tissue shows a reduced catecholamine-induced release of vitamin D [54].

Our results were consistent with some previous studies [24, 25, 55]. A study conducted in 2013 found that Vitamin D insufficiency was significantly associated with elevated uric acid among postmenopausal Chinese Han women but not premenopausal women [24]. Another study conducted by Kamil F. Faridi [55], which involved 4591 adults, was designed to investigate the association between Vitamin D and other non-lipid biomarkers found that adults with deficient 25(OH)D had increased ORs of elevated SUA compared to those with optimal 25(OH)D level. However, there is also some controversial evidence [26, 27, 56]. Two cross-sectional studies conducted by Savas Sipahi [26] and Markus J. Seibel [27], respectively, found that SUA was positively associated with 25(OH)D after adjusting for several potential confounders. Possible explanations of the differences in the above research results included the following: (1) the inclusion and exclusion criteria were diferent among previous research, so there were diferences in the participants, such as Savas Sipahi’s study only involved chronic kidney disease (CKD) patients and Markus J.Seibel’s only involved male participants aged 70 year old and over, which were not representative in normal population; (2) there were differences in the research methods, statistical analysis and adjusted confounding factors, which may also explain the difference.

Our study has some advantages: first, we used a large national representative sample among the general US population which increased the statistical strength to provide a reliable result and we analyzed this association with different statistical methods and fully adjusted the potential confounding factors. We used quartiles of serum vitamin D to explore the relationship between vitamin D and SUA, and we used restricted cubic splines to describe the dose–response relationship between vitamin D and hyperuricemia for the first time. Furthermore, elevated uric acid and secondary hyperparathyroidism are common among CKD patients, so we performed sensitivity analysis by excluding CKD participants to minimize the effect of hyperparathyroidism and renal dysfunction on SUA. Vitamin D, as a fat-soluble vitamin, is lower in the obese people while SUA is higher compared with normal people [50]. To minimize the influence of obesity and fat on the association, we adjusted the BMI, waist circumference, total cholesterol and triglyceride and performed a sensitivity analysis by excluding obesity participants (BMI ≥ 30 kg/m^2^). Although data on the time of sun exposure were not available, we used serum 25(OH)D, which reflected cumulative endogenous synthesis and exogenous supplement, as a direct measure of Vitamin D status to minimize the influence of sun exposure.

However, our study has some limitations. Primarily, as a crosssectional study, it was difficult to determine causality between Vitamin D and SUA. In future, large-scale prospective studies might be required to confirm the causal relationship. Furthermore, due to the limited data, we only analyzed the relationship between SUA and 25(OH)D but did not further analyze the effects of 1,25(OH)D on SUA. In addition, though we performed sensitivity analysis to minimize the influence of PTH, due to lack of PTH data, we could not determine whether the association of 25(OH)D with SUA was partly mediated by PTH and how it mediated. Finally, due to limited data, the dietary regimen information of participants was not involved. Previous studies had proved that different dietary regimens such as ketogenic diets, higher meat, seafood, alcohol and sugar sweetened beverage intake are associated with increased risk of hyperuricemia, while increased Vitamin C intake is associated with decreased hyperuricemia risk [57–60].

It is generally agreed that gout patients should initiate urate-lowering therapy to achieve a specific SUA target, while for asymptomatic hyperuricemia patients, it is not approved in most countries to start uric acid-lowering therapy because of the potential serious adverse effects [61–63]. From this study, for people with unexplained hyperuricemia, it might be necessary to screen serum Vitamin D concentration. Although Vitamin D supplementation has little adverse effects, further studies are needed to explore whether patients with hyperuricemia can benefit from vitamin D supplementation.

## Conclusions

Our study suggests that serum 25(OH)D might be inversely associated with hyperuricemia in general U.S. adults. We hope that it can provide some information for the screening of Vitamin D deficiency and treatment of hyperuricemia. The association we investigated in this study is biologically plausible, and further large-scale prospective studies are required to confirm the causal relationship between SUA and 25(OH)D.

## Data Availability

The datasets supporting the conclusions of this article are available in the public repository as described below. The authors do not own the data. National Health and Nutrition Examination Survey data are available from the National Center for Health Statistics (http://www.cdc.gov/nchs/nhanes/nhanes_questionnaires.htm)

## Abbreviations

NHANES: National Health and Nutrition Examination Surveys
CKD: Chronic kidney disease
SUA: Serum uric acid
25(OH)D: 25-Hydroxyvitamin D
BMI: Body mass index
ORs: Odds ratios
CIs: Confidence intervals
eGFR: Estimation of the glomerular filtration rate
PIR: Poverty income ratio
LC–MS: Liquid chromatography-tandem mass spectrometric
PTH: Parathyroid hormone
